# How will the field of gene therapy survive its success?

**DOI:** 10.1002/btm2.10090

**Published:** 2018-05-24

**Authors:** William F. Kaemmerer

**Affiliations:** ^1^ CGTA Research Group Edina MN

**Keywords:** CAR T‐cell therapies, gene therapies, non‐viral vectors, pay for value, viral vector manufacturing

## Abstract

In August 2017, for the first time, a gene therapy was approved for market release in the United States. That approval was followed by two others before the end of the year. This article cites primary literature, review articles concerning particular biotechnologies, and press releases by the FDA and others in order to provide an overview of the current status of the field of gene therapy with respect to its translation into practice. Technical hurdles that have been overcome in the past decades are summarized, as are hurdles that need to be the subject of continued research. Then, some social and practical challenges are identified that must be overcome if the field of gene therapy, having survived past failures, is to achieve not only technical and clinical but also market success. One of these, the need for an expanded capacity for the manufacturing of viral vectors to be able to meet the needs of additional gene therapies that will be coming soon, is a challenge that the talents of current and future bioengineers may help address.

## 1 INTRODUCTION

The past year was an important one for the field of gene therapy in the United States. The U.S. Food and Drug Administration (FDA) approved the first gene therapy to be marketed in the United States on August 20, 2017 when it provided approval for KymriahTM,^1^ a treatment for acute lymphoblastic leukemia. This was followed shortly by FDA approval for YescartaTM,^2^ a treatment for large B‐cell lymphoma. Both of these are ex vivo gene therapy treatments, in which a transgene is introduced into the patient's own T cells (previously harvested from the patient) outside the patient's body. The purpose of the transgene is to cause the T cells to produce an antigen receptor that will target the T cells to attack the cancer cells. Once these chimeric antigen receptor T cells are successfully transduced and caused to reproduce, then large numbers of these cells are reintroduced into the patient's body to attack the cancer. Toward the end of the year (December 19, 2017), the first in vivo gene therapy for use in the United States received FDA approval.3 Luxturna TM is a treatment for biallelic RPE65 gene mutation‐associated retinal dystrophy. In this treatment, the therapeutic transgene is delivered in vivo into the patient's eye by an intraocular injection. There have been other historic years for the field of gene therapy that have not been as encouraging. In 1999, a trial of a gene therapy for ornithine transcarbamylase deficiency resulted in the death of a patient due to a systemic inflammatory response to the dosage of adenoviral vector that was administered into the patient's bloodstream.^4^ In 2003, it was discovered that the theoretical risk of insertional mutagenesis (in which a viral vector can cause harm to a patient by inserting a transgene into a chromosome in a place that disrupts an existing gene in a detrimental manner) occurred at a much higher rate than expected in children being treated for severe combined immunodeficiency (SCID).^5^ Over time, four out of nine of the children in the initial clinical trial developed leukemia.^6^ Their leukemia was successfully treated, as was their immunodeficiency, but the rate of these detrimental side‐effects, previously expected to have a vanishingly low probability of occurrence, dampened the enthusiasm for gene therapy considerably.

**Figure 1:**
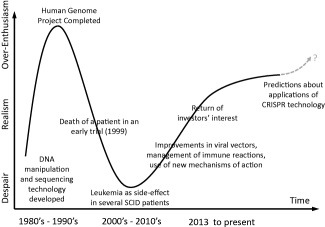
A view of the “hype cycle” the field of gene therapy has traversed

Indeed, the field of gene therapy has undergone the typical cycle of over‐enthusiasm, disillusionment and recovery that Gartner, Inc. analysts have termed the “hype cycle.”^7^ My own description of hype cycle for gene therapy is portrayed in Figure 1. However, if the field has survived its previous failures and emerged from the roller‐coaster ride of the hype cycle, why does the title of this article now pose the question “how will the field survive its *success*?”

This is a review of the current events in the field (cited in press releases) as well as the scientific literature, to consider some challenges that may jeopardize the field's success in the foreseeable future. After enumerating and reviewing the literature regarding the hurdles that the field has mainly overcome, I consider hurdles that are not necessarily major threats to the field's success, but which are still slowing down progress. Then, I consider three more serious issues: the danger of starting a new “hype curve” rise then crash due to over‐promising what results might be quickly achieved by newer gene manipulation technology, the issue of how the cost of gene therapies will be reimbursed, and finally the impending crisis in the mismatch between manufacturing capacity for viral vectors and the emerging therapies that require them. Because many of the therapies nearing approval and all of those that have been market approved so far use viral vectors for delivery of transgenes, this review focuses on viral vectors, particularly adeno‐associated virus (AAV), more than on non‐viral vectors. The review closes by noting ways that bioengineers with an interest in translational medicine may find ways to contribute to solutions to at least the latter issue.

## 2 HURDLES THE FIELD HAS (MAINLY) OVERCOME

Thanks to prior decades' worth of developments in genetic engineering (the use of restriction enzymes, polymerases, ligation agents), the sequencing of the human genome, and the discovery of various ways to influence gene expression (e.g., RNA interference,^8^, ^9^ zinc fingers^10^, ^11^), it is now a straight‐forward exercise to design and build a transgene for the purpose of replacing a missing gene product or reduce the production of a disease‐causing gene product. A variety of ways to “package” one's transgene into a non‐viral or viral vector are now also at bioengineers' disposal, ranging from nanoparticles to a variety of viral vector options (adenovirus, retroviruses, herpes simplex virus, and AAV of various serotypes).^12^, ^13^, ^14^ Long‐term follow‐up periods have established a track record of safety for several viral vectors used for gene delivery in clinical trials.^15^, ^16^, ^17^ Although the actual effects of a given therapeutic agent (transgene plus its delivery vector) versus its intended effects cannot be predicted with precision and remain a matter for empirical testing, one could argue that it is now easier to design a gene therapy vector than it is to discover a new and useful monoclonal antibody or small molecule drug.

That does not mean gene therapy development is easier than drug development. When developing a new drug, one needs to consider the biological target of the drug, its mechanism of action, how the drug affects the body (pharmacodynamics), how the drug is affected by the body (pharmacokinetics) through metabolism, and the rates and routes of elimination. When developing a gene therapy, one must consider all of these, but in addition, how the body will respond to the vector as a potential invader and the transgene product as a foreign entity by mounting an immune response and potentially attacking and eliminating the cells to which the vector has successfully delivered the transgene. For example, earlier attempts at developing gene therapies for hemophilia encountered serious limitations to the efficacy of the therapy due to the lack of persistence in the expression of the transgene caused by a cellular immune response to transduced hepatocytes.^18^ However, this limitation has now been overcome in more recent trials of gene therapies for hemophilia by clinical protocols involving administration of steroids to the patient upon the observation of elevated liver enzyme levels.^19^ At the time of this writing, at least six trials of gene therapy for hemophilia A and seven trials of gene therapy for hemophilia B are listed in clinicaltrials.gov as active and/or recruiting participants. There are published indications that the effectiveness of gene therapy for hemophilia delivered by AAV can be persistent and clinically valuable.^16^, ^20^ Food and Drug Administration (FDA) approval of one or more of these therapies may be achieved within the next year or two. Similarly, a gene therapy for a lysosomal storage disease caused by the deficiency or total lack of an endogenous enzyme for catabolizing a cellular waste product can fail if the patient's immune system reacts to the enzyme produced by the delivered transgene as a foreign protein and mounts a defense, producing neutralizing antibodies or attacking and eliminating the cells expressing the transgene. However, some pre‐clinical research suggests that this limitation can be overcome by techniques for inducing immune tolerance to the transgene product.^21^ Another limitation has been the notion that a viral vector such as a particular serotype of AAV can only be administered to a patient without previous exposure to that serotype, else pre‐existing neutralizing antibodies to the AAV will prevent successful delivery of the therapy. However, this appears not to be an insurmountable limitation. The viral vector for a treatment for hemophilia B being developed by uniQure reportedly has not been limited in effectiveness by neutralizing antibodies to AAV serotype 5.^22^ Some pre‐clinical research has suggested that if re‐administration of the same AAV serotype to the central nervous system of an individual becomes necessary, it might be done without causing an immune reaction if there is sufficient time (11 weeks or more) between the first and second administration,^23^ and re‐administration of the gene therapy for blindness caused by RPE65 mutations (i.e., Luxturna) to the patient's contralateral eye can be performed effectively.^24^ Finally, improvements in lentiviral vectors, which are a type of viral vector in which the delivered transgene is integrated into the recipient's chromosomes, have enhanced the safety of these vectors.^25^

## 3 HURDLES THAT CONTINUE TO SLOW DOWN PROGRESS IN THE FIELD

### 3.1 Delivery hurdles

Of course, there are more technical hurdles to be overcome in the clinical application of gene therapy. A major challenge for most applications is how to deliver or distribute the therapy to enough tissue for efficacy. For chimeric antigen receptor T cell (CAR T‐cell) therapies, this hurdle is relatively low, because the gene therapy agent is delivered ex vivo to cells “in a dish”—a captive audience, so to speak. However, “manufacturing failures” of individual's CAR T‐cell therapies occur, usually due to insufficient harvesting and expansion of the T cell sample from the patient. For the Luxturna gene therapy, the delivery issue is manageable because the viral vector is delivered directly by subretinal injection, using techniques for vector administration that were developed in pre‐clinical research.^26^ Furthermore, the delivery hurdle has a built‐in “divide and conquer” aspect, in that the therapy is delivered to each eye separately.

The hurdle of the distribution of transgene products to replace or supplement enzyme deficiencies in patients is being overcome by the strategy of designing transgene products to be secretable, and delivering the transgenes to the liver with the result that the liver becomes a continuing producer of the enzyme. Aiding in this effort has been the identification of AAV serotypes with particular tropism for the liver^27^ and the use of promoters specific for gene expression from hepatocytes.^28^ The need in many disorders for distribution of transgene products to the central nervous system poses a bigger hurdle, however. Proteins produced and secreted from the liver generally are excluded from distribution to the brain due to the blood‐brain barrier (BBB). For disorders in which transgene delivery to defined parts of brain anatomy can be adequate, direct infusion of viral vectors into the brain tissue by a neurosurgeon is a possible solution. For example, direct injection of an AAV2‐delivered transgene using a surgical approach similar to the implanting of deep brain stimulation electrodes was sufficient for a Phase II trial of a gene therapy for Parkinson's disease to successfully achieve its efficacy endpoint.^29^ An ongoing trial (NCT03065192) of AAV2‐mediated delivery of DNA encoding aromatic l‐amino acid decarboxylase (AADC) for Parkinson's disease is using a direct injection approach involving multiple infusions along a posterior trajectory through the striatum from an occipital entry point. Infusion of AAV vectors into the cerebrospinal fluid (CSF) in the region surrounding the spinal cord (intrathecal delivery) is able to deliver a therapeutic transgene to much of the spinal cord; this approach is being used in an ongoing clinical trial (NCT02362438) for giant axonal neuropathy (GAN).^30^ However, for disorders that may benefit from or absolutely require distribution of the therapy throughout almost all of the brain, the “holy grail” to be sought is a vector capable of delivering a gene therapy across the BBB given that the vasculature is nature's distribution system for reaching the whole brain. AAV serotype 9 has an ability to cross the BBB when administered to the bloodstream,^31^, ^32^, ^33^ but whether this capability is sufficient for “scale up” for human clinical efficacy remains to be seen.^34^ Efforts to re‐engineer the AAV9 serotype to create a novel serotype with much greater BBB‐crossing capability have been successful in mice,^35^ raising the possibility that this hurdle may be overcome through bioengineering. However, a recent report suggests that the means used to engineer the novel AAV serotype in mice may have resulted in a solution that is species‐specific^36^ or even, mouse‐strain specific,^37^ and not transferable to humans. Bioengineers may need to go “back to the drawing board” in this case.

Most daunting of all is the hurdle of how to deliver a transgene to large amounts of the musculature of the human body for the treatment of muscular dystrophy. Despite promising results in mouse models, therapeutic benefits in human trials have not been achieved.^38^ In a proof‐of‐principle trial involving direct injection of AAV into quadriceps muscles in muscular dystrophy patients, no adverse effects were found, but the functional benefit was variable across individuals.^39^ In a canine model of DMD, systemic (intravenous) delivery of AAV9 delivering a mini‐dystrophin gene into neonatal animals resulted in generalized muscle expression of the transgene, but also caused delayed growth, muscle atrophy, and contractures.^40^ More recently, it has been found that intravenous delivery of AAV9 can produce widespread and well‐tolerated muscle transduction of skeletal muscle, diaphragm and heart in a canine model of DMD when the delivery is made in juvenile animals and combined with ongoing immune suppression.^41^ Before translation to human trials, more research will be needed to determine the long‐term safety and efficacy of this approach.

### 3.2 Intellectual property complexity

Another hurdle that is surmountable, but which slows down progress in the field, is the complexity of the intellectual property “territories” that can surround a given gene therapy development. Development and deployment of a new gene therapy entity may involve not only the therapeutic transgene itself, but also its mechanism of action (e.g., RNA interference, CRISPR, etc.), the non‐viral or viral vector used as the delivery agent (e.g., the particular serotype of AAV), and the method for delivery of the gene therapy to the patient (e.g., the delivery devices, surgical techniques, and treatment protocols to be used). While use of some of these therapy components or methods may not involve any patent barriers, others may be protected by patents issued to the original inventors of a specific viral serotype or type of construct used to exploit a mechanism of action (e.g., types of RNA molecules or nucleic acid chemistry for inducing RNA interference). Just identifying what intellectual property owned by what inventor or institution is involved in all the aspects of a new gene therapy can be a complicated task; obtaining license agreements for all the necessary intellectual property holders can consume additional time. As a legal and not a biological matter, there is no bioengineering solution to this problem; however, to the extent that bioengineers' organizations can publicly disclose at least the types of licenses if not the content of the licenses they have obtained for their application, this may allow other developers to save time by following a previously blazed trail through the thicket if they choose to do so.

### 3.3 Clinical trial issues

A number of hurdles slowing the developments of gene therapies are related to how therapies are prepared for clinical trials. It is entirely appropriate for the FDA and other regulatory bodies to require evidence of a therapeutic agent's likely safety with the planned doses and routes of delivery prior to permitting first‐in‐human use. However, although many aspects of a gene therapy may be identical to others that have come before it (e.g., the viral vector, the tissue target, the mechanism of action, the promoter driving the therapeutic transgene, etc.) currently every gene therapy agent or vector is treated as a completely new entity with regards to all the evidence for safety that is required. The National Center for Advancing Translational Sciences (NCATS) of the National Institutes of Health recognized this hurdle recently in a call for information regarding concepts for a “platform” vector that would allow extrapolating clinical pharmacology, safety, or effectiveness assessments from one gene therapy trial to another when the same vector with the same target tissue is used for different disease.^42^ It will be interesting to see what emerges from this call for information in the way of future reports from the NIH or guidance from the FDA.

Another hurdle responsible for slowing the development of some gene therapies is the concern that a gene therapy is inevitably irreversible. Unlike a drug for which the administration can be discontinued or a medical device that can be turned off or explanted, a gene therapy's action may persist for many years, if not for the remainder of the patient's life. This aspect of gene therapy is an advantage with regards to efficacy, allowing for possible “once‐and‐done” treatments, but obviously a disadvantage with regards to safety—“there's no way to turn it off.” As a consequence, regulatory bodies reasonably require pre‐clinical safety testing for a new gene therapy to be extensive and the timing between dose‐escalation cohorts and the length of trials for safety monitoring to be long. In addition, they may require the dosing of the first cohort of patients to be at a conservatively low level with the unfortunate consequence that the first patients receiving therapy are excluded from receiving the potentially greater benefit of a higher dose because, unlike drug treatments, immune response considerations might prohibit re‐dosing of the gene therapy. One may speculate that if a new therapy also had an “antidote” or “safety switch” such that its administration could be reversed at will, then some of the extreme caution regarding first‐in‐human use of the new therapy would be mitigated. There exist some biotechnologies that could provide such a “safety switch” for gene therapies, particularly if implemented in a “platform” vector as called for by the above‐mentioned NCATS call for information.^42^ For example, it has long been known that inserting some particular DNA sequences (loxP sites) into a transgene can allow the intervening DNA between two of these sites to be excised by an enzyme (Cre) delivered either as a protein or as a second transgene. In a pilot study, delivery of a Cre transgene to the cerebral ventricles of a sheep to which a transgene (flanked with loxP sites) encoding for hexosaminidase enzyme had previously been delivered was successfully able to reverse the expression of the first transgene, as evidenced by the return of the newly elevated hexosaminidase enzyme levels in the animal's CSF back to its pre‐treatment baseline.^43^ The emergence of CRISPR gene‐editing technology provides another method that might be exploited for targeting and editing‐out a transgene to reverse a previously delivered gene therapy. The issue from a safety and regulatory standpoint, of course, is whether the delivery of the Cre enzyme or transgene or the CRISPR agent would be totally effective as well as safe in its own right. The undesired persistence of the Cre or CRISPR transgene could be avoided by having that transgene itself contain DNA sequences that would cause the Cre or CRISPR‐Cas action to inactivate it, as well.^44^ Another alternative could be the use of conditional promoters to drive transgene expression. These are promoters that are marginally active in the absence of a co‐factor, such as an orally administered drug (e.g., doxycycline) so that the therapy is only “on” while the patient continues taking the oral medication.^45^ However, the promoter systems available so far are “leaky” rather than absolute in their “off” state, and the drugs that are administered orally to turn on gene expression are not themselves without undesirable side‐effects. Also, the whole system might involve delivery of multiple transgenes, including a regulatory transgene that operationalizes the conditional behavior of the promoter driving expression of the therapeutic transgene, requiring the long‐term safety of the system to be qualified for human use. These technical issues may be solvable; however, today, there may not be any organization with the resources or incentive to develop this platform technology for clinical use for the benefit of the whole field.

Finally, another issue many gene therapy developers must address is how to determine the appropriate size for clinical trials of a therapy for a rare or ultrarare disease, as these are the targets of many gene therapy opportunities. Unless the expected treatment effect is very large, the sample size required for a conventional, randomized controlled trial design could represent a sizeable fraction or even exceed the size of the candidate population, such that the trial is economically unfeasible due to the cost of goods, the cost of patient enrollment and monitoring, and the elimination of patients from a future therapy customer base. Of course, regulatory agencies are not unaware of this issue, and have issued guidelines indicating that “in conditions with small and very small populations, less conventional and/or less commonly seen methodological approaches may be acceptable if they help to improve the interpretability of the study results.”^46^ Resources that can be brought to bear on this issue include the conduct of natural history studies of the relevant patients for use as historical controls and the identification of useful outcome measures,^47^ and the use of one of various alternative clinical trials designs that have been devised^48^ if the design is applicable to an intervention that is irreversible.

The hurdles described above slow down the progress of the gene therapy field, but they do not threaten to “derail it” in a major way. In this author's opinion, there are three other more serious challenges currently facing the field. The answer to “how will the field survive its success?” depends on how well these challenges are met.

## 4 CHALLENGE 1: AVOIDING OVEROPTIMISM

As described by Fenn and Raskino^7^ in their discussion of the “hype cycle,” any sufficiently new and potentially powerful technology produces an initial reaction among participants and onlookers of optimism regarding what the new technology will allow us to accomplish. This was the case with gene therapy in combination with the completion of the Human Genome Project in the late 1990s when it was thought that the power of genetic engineering coupled with the new knowledge of the entire human genome sequence would quickly lead to treatments, if not cures, for many diseases. When these treatments failed to materialize and problems emerged, interest in gene therapy development waned among investors in small companies, and large companies dismissed gene therapy as something “always five years away, year after year” and therefore not meriting their investment. However, since about 2012, the interest in gene therapy by investors has returned. Venture capital companies have supported numerous start‐ups (Spark Therapeutics, Voyager Therapeutics, Bluebird Bio, Wave Therapeutics, Dimension Therapeutics, Bamboo Therapeutics, etc.) Some of these companies have already been acquired by large pharmaceutical companies or other corporations; others have attracted significant funding via partnership deals. While all of this is good news for the field, there is a possibility that some investors' enthusiasm is becoming too high. For example, some analysts have suggested that a potential gene therapy for diabetic neuropathic pain, peripheral artery disease and ALS could be worth over $9.3 billion U.S. dollars assuming just 15% market penetration.^49^ This valuation must involve the assumption of a considerable revenue from the ALS indication, because it exceeds the valuation of the *entire* global diabetic and peripheral neuropathic pain market others have estimated to reach $8.3 billion in the year 2024.^50^ CRISPR‐Cas gene‐editing technology, itself perhaps subject to following a “hype curve” over the next several years, may affect attitudes toward the field of gene therapy. Despite having “nearly limitless potential to do real good in the world,”^51^ delivery of many CRISPR‐based therapies may rely on the same viral vectors (e.g., AAV) as other gene therapies and face the same limitations.

A reason for caution and a reason for considering this challenge to be more serious for the field than the other “hurdles” is the time that can be required for investors' interest to return, once they are disappointed. Hurdles slow down progress while researchers work to overcome them; pendulum swings in investor attitudes can result in long seasons of “winter” for a field. A case in point: coincident with the discouragement that set in after the setbacks in gene therapy with the death of a clinical trial participant in 1999 and higher than predicted incidence of insertional mutagenesis causing leukemia in SCID patients receiving retroviral vectors, it was 13 years before the number of IND submissions to the FDA for gene therapy trials in the United States returned to and eventually surpassed the number submitted in 1999. Some reason for this time lag and then recovery may have been the time required for further enabling technologies to emerge from laboratories to clinical readiness; however, a “drought” in investors' interest in gene therapy in the 2000s was likely also a factor.

A second reason for caution is that the limitations of gene therapy vectors for treatments requiring distribution to the entire body, as some gene‐editing treatments might require, are still being learned and may not always be appreciated. For example, a recent experiment in rhesus monkeys found that the high doses of AAV that may need to be delivered intravenously for some therapies could be prohibitively toxic.^52^ In three out of three primates and three out of three piglets, the investigators found that the high doses of AAV used resulted in severe toxicity, including hepatocellular necrosis and axonopathies in both the central and peripheral nervous system of the monkeys, and neuronal degeneration in the dorsal root ganglia of the piglets. In commenting on this report, Flotte et al.^53^ noted that the doses used in the animals with these serious outcomes are nevertheless being used in human trials that so far have not encountered dose‐limiting toxicity, and that the toxicity encountered in the primates and piglets could have been due to some unidentified contaminants in the viral preparation. They concluded that the field must neither ignore nor overreact to these findings regarding the toxicity of high systemic doses of AAV.

## 5 CHALLENGE 2: IMPLEMENTING REIMBURSEMENT INNOVATIONS

A second major challenge to the field is the issue of how gene therapies that have achieved success in clinical development will achieve success in the marketplace. A case in point is that of Glybera™, a gene therapy for lipoprotein lipase deficiency developed by uniQure, Inc., and approved for marketing in Europe in 2012—the first gene therapy to receive market approval in the Western hemisphere. Although some reviewers questioned whether there was strong enough efficacy data for regulatory approval,^54^ nevertheless, it was determined that this single‐treatment therapy does provide benefit to patients, particularly by reducing the occurrence of pancreatitis, which can be life‐threatening. It was marketed in Europe in 2015 at a price equivalent to US $1 million,^55^ a cost comparable to about 3 years' worth of enzyme replacement therapy (at $360,000 per year) for other enzyme deficiency disorders.^56^ While this inherited disease is rare, it is not ultrarare; its prevalence is approximately 1–2 million persons worldwide. Even so, in 2016, only one patient was treated post‐market release of the therapy, and in 2017, uniQure elected not to renew its market authorization for the product.

GlaxoSmithKline (GSK) has had a similarly difficult time with their gene therapy (Strimvelis) for SCID due to adenosine deaminase deficiency. In this case, the evidence for the efficacy of the treatment and the magnitude of the benefit is overwhelming. One dose of the therapy provides what is essentially a cure for the disease—there was a survival rate of 100% of the 18 children involved in the clinical trials of the treatment.^57^, ^58^ The therapy was priced at 594,000 Euros in 2016, with GSK also providing a “money‐back guarantee.” Nevertheless, as of 2017, only two patients had received the treatment with two others “in queue” to receive the therapy, and GSK was seeking a buyer for Strimvelis.^59^

What is the problem? Are parents not motivated to save the lives of their children? Are the treatments too painful or too onerous to be tolerated? Have companies not sufficiently publicized the availability of these therapies? Are companies setting prices too high out of pure greed? None of the above. The problem is twofold.

First, the attractive property of many gene therapies (that they can be “once and done” lifetime treatments) coupled with the other property that the treatments are expensive not only to develop but also to provide (with a high “cost of goods”) requires a high price for the therapy for its production to be economically viable. Unlike small molecule drug therapies that are expensive to develop but later less costly to manufacture, or enzyme replacement therapies that are expensive to develop and costly to produce but are not one time treatments, gene therapies do not intrinsically provide an ongoing revenue stream to the producer to recoup costs and make a profit over time.^1^ Unlike “once and done” therapies based on capital equipment such as cardiac ablation equipment acquired by hospitals, the up‐front investment cost (revenue to the manufacturer) is not amortized over time and multiple patients by the provider. Instead, the revenue needed must be derived from the patient's first and perhaps only treatment.

Second, our current systems for paying for treatments are not at all well‐suited to these “front‐loaded” costs. New reimbursement models are needed.

### 5.1 The mismatch of once‐and‐done treatments and reimbursement models

Currently, in the United States, reimbursement rates tend to be based on the cost of comparable procedures performed by the health care provider in delivering treatments, plus the cost of goods and other supplies and equipment provided by the clinic or hospital. This model does not work well for a therapy for which the procedure costs are only modest (e.g., placing an intravenous line and performing an infusion) but the costs of goods is high but not‐recurring. Necessarily, then, the lion's share of the reimbursement must be based on weighing the cost of goods against the benefit to the patient, moving the model from “pay for procedure” to “pay for value.” The issue then becomes how to place an economic value on an individual's life to decide what is a justifiable price and equitable reimbursement rate for a life‐saving treatment. As daunting and onerous as this type of calculation may seem at first glance, it has been tackled before by health economists, and standards have emerged for deciding whether expected benefit of a therapy measured by such metrics as “quality adjusted life years” is sufficient to justify a proposed price. A report by the Institute for Clinical and Economic Review prepared for the California Technology Assessment Forum exemplifies the kind of work involved, presenting an extensive analysis estimating the cost‐effectiveness of CAR T‐cell therapies for the treatment of B‐cell malignancies.^60^ The report includes, for example, an analysis for the treatment of pediatric relapsed/refractory B‐cell acute lymphoblastic leukemia patients with tisagenlecleucel (Kymriah™, Novartis), indicating that a net price including hospital markup of $575,000 paid for responders at one month compares favorably with the threshold needed to achieve a rate of $100,000 per Quality Adjusted Life Year of expected benefit in these patients, one commonly used cost‐effectiveness threshold.

Unfortunately, moving to the “pay for value” model for health care reimbursement still does not solve the problem of how the high up‐front cost of a gene therapy can be afforded by payers, whether individuals or third‐party payers. Recognizing this, companies producing gene therapies are now seeking other innovations, such as moving from “pay for value” to a “pay for outcome” model. Novartis, provider of Kymriah, is taking this approach, offering the therapy on a “delayed payment” basis whereby payment for the treatment only is required after evidence that the therapy has worked, and the patient's cancer has gone into remission. How many months must elapse after treatment for the patient's health status to qualify as “remission” then becomes a point of debate.^61^ However, “pay for outcome” alone may not be a solution to the problem of reimbursement for a gene therapy, as the failure of GSK's “money‐back guarantee” to encourage greater adoption of Strimvelis showed.

Delayed payment may be as much a barrier to adoption as immediate payment, unless the reimbursement for the therapy can be spread over time. “Payment for continued benefit” is an innovative approach that provides for such amortization. In this approach, a recipient of a gene therapy (or their insurance company) would pay for the therapy they received a year at a time, as long as the patient continues to benefit from the therapy year after year. However, besides having the unintended effect of providing an incentive for a patient or their payer to show (or feign) poor health in order to discontinue payments, this approach is still a mismatch to health care systems in the United States in which patients are used to being able to change insurance companies at will on an annual basis. Even more so than the contentious issue of coverage for pre‐existing conditions, a payment scheme that spreads the up‐front cost of a gene therapy over time may lead to controversies over whether insurers must provide coverage for persons with “pre‐existing payment obligations.” Nations with single‐payer systems (universal health coverage provided by a government agency) may be better able to adopt this approach, and cover gene therapies despite their high cost, knowing that their system will also reap the future benefits of the cost‐avoidance (e.g., for expensive palliative and end‐of‐life care) that an effective “once and done” treatment may provide.

It is apt that the leadership of the gene therapy company with the ticker symbol “ONCE” (Spark Therapeutics, Jeff Mazzarro, CEO) has recognized the need for innovations in reimbursement schemes for the field of gene therapy to be successful and is joining several other leaders in health care in work to meet this challenge.^62^

## 6 CHALLENGE 3: MEETING THE LOOMING MANUFACTURING DEMAND

A third major challenge to the field's continued success, at least in the near term, is the challenge of developing the manufacturing capacity of the United States and other countries sufficiently to meet the coming demand for the therapeutic agents, specifically AAV production. This has not been a major challenge so far because the therapies approved have been ones for which the amount of vector needed to treat a patient is relatively small. The amount of viral vector needed for a CAR T‐cell therapy is only the amount needed for effective ex vivo transduction of the patient's T cells in a cell culture setting. The amount of viral vector to treat a patient via intraocular injection is only on the order of 10^11^ vector genomes per injection, a small amount relative to current state‐of‐the‐art manufacturing capabilities.

In contrast, other therapies on the horizon will require orders of magnitude more viral vectors for the dosing of individuals. Currently, trials for gene therapy for hemophilia A are underway by Biomarin, Sangamo, Shire, and Spark Therapeutics, and trials of gene therapy for hemophilia B are underway by AskLepios Bio, Dimension Therapeutics (acquired by UltraGenyx), Sangamo, and uniQure—and this may not be an exhaustive list. There are indications that the effectiveness of a gene therapy for hemophilia can be persistent and clinically valuable.^19^, ^22^ FDA approval of one or more of these therapies may be achieved within the next year or two. An idea of how much vector manufacturing capacity these therapies will require for sufficient supply of products for market success can be seen by noting the dosage expected per patient, compared to a vector genome yield that is feasible given the current state‐of‐the‐art in AAV manufacturing, which is about 1 e^16^ vector genomes per manufactured lot (see Table 1).

**Table 1:** Approximate amount of AAV required for various therapies

Therapy or indication	Route of delivery	Approximate AAV dose per patient (vector genomes)	One manufactured lot of 1 e^16^ AAV would treat how many patients?
Luxturna/Leber's congenital amaurosis/(Spark Therapeutics)	Intraocular injection	3.0 e^11^	33,000
VY‐AADC01/Parkinson's disease/(Voyager Therapeutics)	Direct injection into brain tissue	4.7 e^12^	2,127
Hemophilia	Intravenous	6 e^13^/kg (∼3.6 e^15^ for a 60 kg individual)	3
Muscular dystrophy	Multiple injections into muscles	1 e^14^ / kg (∼6.0 e^15^ for a 60 kg individual)	2
Lysosomal storage disorders	Intravenous and/or into cerebrospinal fluid	2.5 e^15^	4

As indicated in Table 1, whereas a single GMP lot of AAV that is qualified and released for market use might be sufficient inventory for 33,000 patients receiving an intraocular gene therapy, the same lot may only be sufficient inventory for three patients receiving an intravenous dose of AAV to treat their hemophilia. Even though these therapies are not yet market approved, the production demand posed by the need for sufficient AAV for the conduct of clinical trials has already resulted in long queues for contract manufacturing services, delaying by months to years the development of other therapies by organizations with no manufacturing capabilities of their own. Several companies have foreseen this problem looming on the horizon. The approach of several therapy developers has been to seek an internal manufacturing capability. For example, uniQure has established its own manufacturing facility in Lexington, Massachusetts. Pfizer has acquired Bamboo Therapeutics,^63^ a spin‐off of the University of North Carolina vector core^64^ to enhance AAV production capacity (which had the effect of removing some smaller organizations' access to this capacity) and they are proceeding to invest in the building of additional capacity.^65^ Contract manufacturing companies are also seeing the future increased demand for viral production and are working to build more capacity. For example, Brammer Bio has recently doubled its capacity in Alachua, Florida^66^ and renovated a 66,000 square foot facility in Cambridge, Massachusetts.^67^ However, increasing capacity is not just a matter of adding square footage and clean rooms—there is also a need for expertise and experience, which will take time to acquire.

### 6.1 Non‐viral vectors to the rescue?

An option that may partially solve this manufacturing capacity shortfall, as well as provide other features for gene therapies that viral vectors do not provide, will be the use of non‐viral vectors, that is, nanoparticle or other delivery formulations for delivering DNA to patient's cells, in vivo or ex vivo. See Ramamoorth et al. 2015^68^ and Jayant et al. 2016^69^ for reviews of the state‐of‐the‐art in the development and application of non‐viral vectors for the delivery of gene therapies. The advancement of non‐viral vectors to use in clinical trials has lagged that of viral vector usage, but in the future non‐viral systems may be complementary to viral‐based delivery systems. For example, Generation Therapeutics (https://generationbio.com) is company founded recently to develop therapies using closed‐ended, linear duplex DNA technology.^70^ It is hoped that this biotechnology will allow the development and clinical deployment of therapies for such indications as progressive familial intrahepatic cholestasis, phenylketonuria and glycogen storage disease 1a, and allow the delivery of the therapies to the liver to be titrated to best clinical effect in a growing patient, a capability that viral vectors do not provide. Similarly, Stoke Therapeutics (https://stoketherapeutics.com) is a company seeking to develop antisense oligonucleotide therapeutics delivered without the use of viral vectors to treat diseases through a mechanism of action involving affecting the exon splicing of mRNA to enhance the production in cells of effective mature mRNA molecules.^71^ It is possible for chemically synthesized oligonucleotides to be delivered as drugs, even as “naked” DNA, and be taken up by cells. For example, direct infusion of naked siRNA molecules into the putamen in non‐human primates results in their uptake and the induction of RNA interference in cells.^72^, ^73^

Nevertheless, despite some advantages of manufacturing and using non‐viral vectors compared to viral vectors for delivery of gene therapies (see Table 2), there are other features of viral vectors that non‐viral vectors do not match, the foremost being the limited transduction efficiency of non‐viral vectors compared to viral vectors.^74^

**Table 2:** Relative pros and cons of viral versus non‐viral vectors

Consideration	Viral vectors	Non‐viral vectors
Transduction efficiency	Comparatively good	Comparatively poor; a key limitation for non‐viral vectors.
Persistence of expression	Years and perhaps patient's lifetime; double‐edged sword vis‐à‐vis reversibility.	Generally shorter than with virally administered transgenes; repeated dosing will be required except for mechanisms of action that are permanent (e.g., gene editing)
Reversibility of effect	Not clinically possible, although technical solutions exist that could be developed, depending on mechanism of action (e.g., protein replacement vs. permanent gene editing mechanism).	A strength for the use of non‐viral vectors, depending on mechanism of action (e.g., protein replacement vs. permanent gene editing mechanism).
Ability to titrate dose to effect in patient	Not possible; dose required for effectiveness is difficult to predict; requires applications with a large therapeutic window between the minimally effective dose and the maximally tolerated dose.	A strength for the use of non‐viral vectors, although relationship between dose and effect must be empirically established.
Possibility for repeated dosing	Immune response to first dosing may limit effectiveness or prohibit use of an additional administrations of the same viral serotype.	Comparatively better, though an immune response to novel transgene product may still pose a limitation.
Risk of insertional mutagenesis	Not an issue for AAV; minimized in newer generations of lentivirus.	Non‐existent to minimal, depending mechanism of action (e.g., transposons can insert DNA into unpredictable host chromosome locations).
For diseases with central nervous system (CNS) involvement:		
CNS distribution via axonal transport	Comparatively good to excellent (a feature of many AAV serotypes).	Comparatively poor to non‐existent.
Neuronal specificity	A feature of some AAV serotypes; can be useful for avoiding immune response in nervous system mediated by glial cells.	Carriers with neuronal specificity remain to be developed.
Crossing of the blood‐brain barrier	A feature of some AAV serotypes; further developments needed for clinical utility.	Requires nanoparticles with peptides or other conjugates for uptake across BBB; decades of research have not yet yielded clinically deployable solution.

As with viral vectors, the development of processes and manufacturing capacity for producing clinical grade non‐viral vectors to scale will also require significant effort. If the therapeutic application involves delivery of lengthy molecules of DNA rather than chemically synthesizable oligos, then the manufacturing of non‐viral vectors will benefit from bioengineering developments in the first step in viral vector manufacturing, namely the GMP production of plasmids. Successful clinical deployments of gene therapies based on non‐viral vectors are at least several years away, and then, they will be unlikely to displace the role of viral vectors in the marketplace. Therefore, improvements in technology that improve the yield of viral vector manufacturing will remain an important area for bioengineers' attention.

### 6.2 An example of the manufacturing processes involved: production of AAV

**Figure 2:**
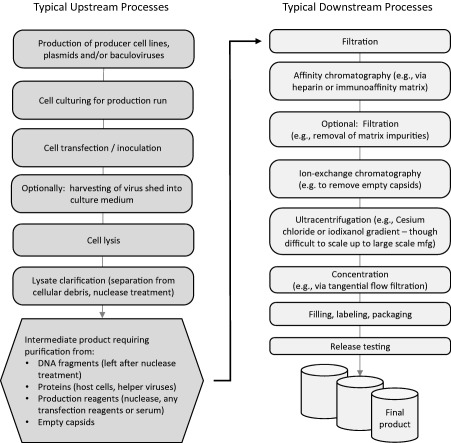
Typical steps in the manufacturing of an AAV product

The production of AAV involves multiple “upstream” and “downstream” processes. An overview of the process steps in a typical AAV manufacturing protocol is portrayed in Figure 2.

Note that not all of the steps shown in the overview figure are included in every manufacturing protocol. Also, note that there are alternative choices to be made for most of the steps. For example, AAV can be produced in mammalian cells, such as HEK293 cells, transfected by multiple plasmids with one containing the transgene of interest and others providing the DNA encoding for the capsid proteins and the helper genes that must be present before AAV will be produced in the cells. Alternatively, AAV can be produced in insect cells transduced with baculoviruses providing these constructs,^75^, ^76^ an advancement in technology that has increased the yield of AAV from cells in the upstream part of the production. For a more detailed overview of the upstream aspects of AAV production, see the review by Penaud‐Budloo et al.^77^

Unfortunately, the encouraging amount of AAV yield that can be achieved in the upstream steps, as high as 2 × 10^5^ vector genomes per cell, is considerably reduced by the downstream steps required for vector purification. The intermediate material obtained from lysis and clarification of the lysate from the producer cells contains many classes of materials and contaminants that must be removed to yield the final, purified product. These include residual DNA fragments, host cell proteins, residual transfection reagents, and empty capsid particles. The problem is that there is currently no technology capable of removing all of these contaminants and yielding the purified product in a single step. For example, affinity chromatography can separate AAV particles from other proteins, but cannot discriminate between empty and full AAV particles. Ion exchange chromatography can separate full from empty capsids, but it requires careful “tuning” of the process conditions (pH, salt content, etc.) to be successful.

Since in the downstream part of AAV production multiple process steps need to be performed in series to purify the product, clearly even if each step is 75% efficient resulting in only 25% loss of yield due to that step, it only takes a series of three steps at this efficiency to reduce the overall yield of the final product to 42% or even less, considering that some of the yield that must be devoted to in‐process and final product safety, purity, and quality testing requirements.

Research is needed to improve both the upstream and downstream steps of AAV production. Bioengineering can contribute to improvements in the upstream yield of AAV through the engineering of improved bioreactor equipment. For a review and analysis of how improvements in downstream steps might be made based on the consideration of the properties of AAV, including size, mass, isoelectric point, and other physicochemical properties, see Qu et al.^78^

When producing AAV for use in pre‐clinical research, and particularly when producing the amount of AAV needed for studies in small animals, the loss of more than half of the amount of vector produced to the processing steps usually did not have a major impact on the availability or cost of AAV to the researcher. Now, however, a loss of more than half of the yield of a manufacturing lot of vector equates to more than a doubling of the cost of goods of the product to the clinical trialist, and ultimately to the patient receiving the marketed therapy. Therefore, process improvements and perhaps break‐through innovations are needed—worthwhile for bioengineers to devote their time, energy, expertise, and creativity to find and develop.

## 7 CONCLUSION: IMPLICATIONS FOR BIOENGINEERING AND TRANSLATIONAL MEDICINE

Finally, after decades of work, scientists, translational researchers, and clinicians are seeing the fruits of their dedication in the form of market‐released gene therapies for relapsed cases of leukemia and inherited forms of blindness, bringing new hope to patients who previously had few or no options for longer life or better quality of life. Now, while research and development efforts to tackle the issues of delivery, immune rejection, and scalability of gene therapy continue, additional efforts are needed to insure that the field of gene therapy can “survive its success.” Although bioengineers may have limited ability to prevent over‐enthusiasm or reimbursement issues from threatening the field's continued success, they have a role to play in reducing the cost of gene therapies and tackling the challenge of enhancing manufacturing capacities to meet the demands of emerging therapies. They can bring their expertise and creativity to bear on the problem of how to improve manufacturing yields for viral vectors, and future non‐viral vectors as well.

## CONFLICT OF INTEREST

The author is a co‐founder of the CGTA Research Group, serves as science advisor and statistician to the New Hope Research Foundation, a non‐profit organization developing a gene therapy for GM2 gangliosidoses, and is a member of the scientific advisory board of Alcyone Lifesciences, Inc., a company that produces catheters for intracranial use. The opinions expressed in this review are those of the author alone, and the author has no conflicts of interest to declare.

## NOTE

1 An attempt to generate an ongoing revenue stream by using a conditional promoter to drive a therapy, then charging for a proprietary oral drug to keep the therapy on (even if such a system could be deployed) would likely be unfavorably viewed by consumers, and circumvented by a black market in the oral drug, which would eventually go off‐patent anyway.
